# COVID-19 Prevention and Treatment

**DOI:** 10.3390/life13030834

**Published:** 2023-03-20

**Authors:** Silvia De Francia, Francesco Chiara, Sarah Allegra

**Affiliations:** Department of Clinical and Biological Sciences, University of Turin, Regione Gonzole 10, 10043 Orbassano, Italy

## 1. Introduction

Coronavirus disease 2019 (COVID-19) has spread and become a substantial public health concern worldwide. COVID-19 is an infectious disease caused by the recently discovered SARS-CoV-2 virus. This new virus and the associated disease were unknown before the outbreak reported in Wuhan, China in December 2019. The incubation period for COVID-19 is 1–14 days, most commonly around 5–7 days. The disease causes respiratory illness with symptoms such as a cough, fever, tiredness, and, in more severe cases, difficulty breathing. The COVID-19 virus may persist on surfaces for a few hours up to several days and this may vary under different conditions, e.g., the type of surface, temperature, or humidity of the environment. COVID-19 was first described as a respiratory disease, but presently it is considered a systemic infection comprising multiple systems and causing chronic complications [1]. The pathology results not only from viral infection but from an aberrant inflammatory host immune response [2]. The immune response has been well described in acute COVID-19 patients, but the lasting consequences of the infection are still not well known. The principal mode by which people are infected with SARS-CoV-2 is through exposure to respiratory fluids carrying the infectious virus. Exposure occurs in three principal ways: (1) inhalation of respiratory droplets and aerosol particles; (2) deposition of these particles on exposed mucous membranes in the mouth, nose, or eye by direct splashes and sprays; and (3) touching mucous membranes with hands that have been soiled either directly by virus-containing respiratory fluids or indirectly by touching surfaces with virus on them [3]. The infectious dose of SARS-CoV-2 needed to transmit infection has not yet been established. Current evidence strongly suggests transmission from contaminated surfaces does not contribute substantially to new infections. The risk of SARS-CoV-2 transmission can be reduced by covering coughs and sneezes and maintaining distance from others. When consistent distancing is not possible, well-fitted masks may reduce the spread of infectious droplets from individuals with SARS-CoV-2 infection to others. Frequent hand washing also effectively reduces the risk of infection [4]. Health care providers should follow the Centers for Disease Control and Prevention (CDC)’s recommendations for infection control and the appropriate use of personal protective equipment [5]. At the end of 2021, COVID-19 vaccines received approvals for human use in several countries worldwide. Vaccination is the most effective way to prevent COVID-19. The COVID-19 Treatment Guidelines Panel recommends COVID-19 vaccination as soon as possible for everyone who is eligible according to the CDC’s Advisory Committee on Immunization Practices. Four vaccines are authorized or approved for use in the United States to prevent COVID-19. For primary and booster vaccinations, the mRNA vaccines BNT162b2 (Pfizer-BioNTech) and mRNA-1273 (Moderna) and the recombinant spike protein with matrix-M1 adjuvant vaccine NVX-CoV2373 (Novavax) are preferable to the adenovirus vector vaccine Ad26.COV2.S (Johnson & Johnson/Janssen), because of the latter’s risk of causing serious adverse events [6]. Reports have suggested that there is an increased risk of thrombosis with thrombocytopenia syndrome (TTS) in adults who received the Johnson & Johnson/Janssen vaccine [7] and, rarely, the Moderna vaccine [8]. TTS is a rare but serious condition that causes blood clots in large blood vessels and low platelet levels. The American Society of Hematology has published considerations relevant to the diagnosis and treatment of TTS that occurs in people who receive the Johnson & Johnson/Janssen vaccine [9]. These considerations include information on administering a non-heparin anticoagulant and intravenous immunoglobulin to these patients. Pregnant and lactating individuals were not included in the initial COVID-19 vaccine trials. However, the CDC, the American College of Obstetricians and Gynecologists, and the Society for Maternal-Fetal Medicine recommend vaccination for pregnant and lactating people based on the accumulated safety and efficacy data on the use of these vaccines in pregnant people [10,11]. These organizations also recommend vaccination for people who are trying to become pregnant or who may become pregnant in the future. As of March 2023, there are three Food and Drug administration (FDA)–authorized antiviral treatments available to treat people who have mild or moderate symptoms of COVID-19 infection and have risk factors for severe illness. These treatments have been shown to reduce the risk of hospitalization and death in this population: a combination of nirmatrelvir and ritonavir (Paxlovid), molnupiravir (Lagevrio), remdesivir (Veklury). These drugs work by targeting specific parts of the SARS-CoV-2 virus to stop it from multiplying in the body. Another treatment that may benefit immunocompromised people with COVID-19 infection is convalescent plasma. As of March 2023, tocilizumab (Actemra) is the only monoclonal antibody treatment with FDA emergency use authorization (EUA) to treat severe COVID-19 infection, helping to prevent complications. As of March 2023, in case of mild symptoms of COVID-19, to reduce fever and ease aches and pains, acetaminophen and ibuprofen are recommended [12].

The Special Issue “COVID-19 Prevention and Treatment” published in *Life* (ISSN 2075-1729), belonging to the section “Epidemiology”, collects a series of research and review articles related to better design routes of prevention and treatment for COVID-19. This issue contains 21 articles. The following is an overview divided by themes addressed by the manuscripts.

## 2. Treatment and Prevention

Eleven of the papers focus on one of the most important topics related to the COVID-19 pandemic: how to prevent and how to treat the disease. Seven of them are focused on treatment and four of the papers focus on prevention. Three of the papers about treatment are related to the use of convalescent plasma, considered for its immunological mechanisms as benefit for patients in moderate and severe stages of COVID-19. The studies evaluated the safety and efficacy of its use [13,14,15]. As of December 2020, when the therapeutic agents approved for COVID-19 were limited all over the world, plasma from individuals recovered from COVID-19 was the first therapeutic tool adopted. Another four papers addressing treatment are related to the potential therapeutic implications of different substances or models [16,17,18,19]. The studies covered the use of Renessans, a product with iodine complexes and ascorbic acid (the study was designed to determine its efficacy for SARS-CoV-2 in *Rhesus macaque*), characterized by antimicrobial activity; the use of oxygen (the study clearly underlines how, in patients presenting with early dyspnea, the primary goal of therapy should be the reversal of brain hypoxia with a first approach of intermittent treatment with 100% oxygen using a tight oronasal mask or a hood); the use of 14 Mev neutron irradiation (neutron radiation is usually used to sterilize viruses because neutron radiation is 10 times more effective than gamma rays in inactivating viruses: the authors established a closed SARS-CoV-2 inactivation container model and simulated the inactivation performance by using several different neutron sources); and the application of lung imaging scores (the main goal of the paper was to propose a prediction model involving imaging methods, specifically ultrasound). Four of the eleven papers are focused on prevention, the use of vaccines, and their complications [20,21,22,23]: the VeroCell vaccine (in peruvian health workers), three different vaccination schedules administered in Chile until January 2022, the potential neurological complications of vaccines, and KD-414 as a booster vaccine for SARS-CoV-2 in healthy adults (KAPIVARA).

## 3. Mechanism of Action

Two of the papers of the Special Issue focus on potential mechanism of action involved in COVID-19’s pathogenicity [24,25]: one paper explored the role of krebs von del Lungen-6 in severe-to-critical COVID-19 patients (KL-6 is a glycoprotein expressed mainly from type II alveolar cells with pro-fibrotic properties: serum KL-6 concentrations have been found in patients with COVID-19); and the second one showed potential therapeutic implications of how COVID-19 hijacks the cytoskeleton (after attaching to membrane receptors and entering cells, the SARS-CoV-2 virus co-opts the dynamic intra-cellular cytoskeletal network of microtubules, actin, and the microtubule-organizing center, enabling three factors that lead to clinical pathology: viral load due to intra-cellular trafficking, cell-to-cell spread by filopodia, and immune dysfunction).

## 4. Health System Organization

Three of the papers of the Special Issue focus on health system organization involved in COVID-19 management [26,27,28]: one paper explored the potential role of sex and gender differences in COVID-19 management (the virus mainly affected men with worse symptomatology due to a different immune system, which is stronger in women, and to the Angiotensin-converting enzyme 2 and Transmembrane protease serine 2 roles, which are differently expressed among the sexes; additionally, women are more inclined to maintain social distance and smoke less). The main objective of second study was to describe the measures taken to provide optimal medical care to patients who presented themselves in one of the large emergency hospitals of Romania in the fourth wave of the COVID-19 pandemic, and the third study was aimed at evaluating the government of Nepal’s response to the COVID-19 pandemic.

## 5. General and Specific Condition of Pathology

Five of the papers of the Special Issue focus on the general and specific conditions of COVID-19 pathology [29,30,31,32,33]: one paper explored the potential role of inflammation as a prognostic hallmark of clinical outcome in infected patients. The main objective of a second study was to describe the role of host genetic variability in modulating COVID-19 clinical outcomes. The third study evaluated post-COVID-19 conditions in terms of complications, adverse events, and risk factors. Another paper explored the association between asthma and COVID-19 (interestingly, asthma characterized by type 2 inflammation displays a cellular and molecular profile that may confer protective effects against COVID-19) and the last one was a case report about a 69-year-old man with known seropositive generalized myasthenia gravis, hypertension, ischaemic heart disease, cerebrovascular disease, and recurrent urinary tract infections. He was admitted to the ICU for mixed acute respiratory failure, elevated serum lactate and liver function enzymes, and severe thrombocytopenia and a SARS-CoV-2 PCR test was positive.
